# Playfulness from children’s perspectives: development and validation of the Children’s Playfulness Scale as a self-report instrument for children from 3 years of age

**DOI:** 10.3389/fpsyg.2023.1287274

**Published:** 2023-12-05

**Authors:** Isabelle Duss, Cornelia Rüdisüli, Corina Wustmann Seiler, Patricia Lannen

**Affiliations:** ^1^Marie Meierhofer Children’s Institute, Associated Institute of the University of Zurich, Zürich, Switzerland; ^2^Zurich University of Teacher Education, Zürich, Switzerland

**Keywords:** playfulness, play, children, self-report, self-concept, validation, multiple-group invariance testing

## Abstract

Children’s playfulness refers to children’s enjoyment, motivation, and engagement in play and has been predominantly assessed from an adult perspective. To assess children’s perspectives on their own playfulness, we adapted and modified the Children’s Playfulness Scale (CPS) for children from 3 years and used a two-level response format with a total of four answer options. We tested the self-report scale with 564 children between 3 and 8 years of age who attended childcare center or kindergarten. Results indicated that the adapted version of the CPS identified the five distinct domains of playfulness: social spontaneity, cognitive spontaneity, physical spontaneity, sense of humor, and manifest joy; furthermore, results showed invariance across multiple groups for gender, age, and language skills. A highly significant positive correlation was found between children’s self-reported playfulness and children’s self-reported social self-concept (*r* = 0.54, *p* < 0.001), which demonstrates convergent validity. No association was found with teacher proxy report of children’s playfulness (*r* = 0.03, *p* = 0.92). Overall, our study confirmed first indications of the validity of the modified CPS as a reliable instrument for assessing children’s self-reported playfulness. This enables children as young as 3 years old to be assessed on their own playfulness, which is a valuable supplement to the adult perspective.

## 1 Introduction

Professional and academic consensus has long recognized the essential role of play in child development (Ginsburg, 2007; Whitebread et al., 2017). Through play, young children explore their surroundings, develop various skills, and increase their knowledge (Vygotsky, 1967; Piaget, 1976). Playing and learning occur simultaneously in young children (Zosh et al., 2017; Nilsson et al., 2018). Consequently, children’s enjoyment, motivation, and engagement in play, also referred to as playfulness, are seen as particularly vital for children’s development independently of the play activity itself, such as construction play and symbolic play (Bundy, 1997; Cornelli Sanderson, 2010). For example children’s playfulness has been found to be related to their coping skills, motor flexibility, and emotion regulation (Saunders et al., 1999; Trevlas et al., 2003; Ruckser-Scherb, 2010; Christian, 2012).

Children’s playfulness has been assessed almost exclusively from an adult perspective (e.g., Trevlas et al., 2003; Pearton et al., 2014; Yoon et al., 2014; Waldman-Levi et al., 2015; Keleş and Yurt, 2017; Pinchover, 2017). To date, only one study has examined children’s playfulness from their own perspective (Fink et al., 2020). However, Barnett (2013) showed that children’s responses to their play behaviors differ substantially from those of adults. This indicates the importance of capturing the perspectives of the children themselves.

This study adapted and validated the Children’s Playfulness Scale (CPS; Barnett, 1991) for children from 3 years with a multidimensional structure underlying the construct and operationalization of children’s playfulness.

### 1.1 Play and playfulness in early childhood

A growing body of evidence links play to children’s physical, social, and emotional well-being (e.g., Ginsburg, 2007; Lillard et al., 2013) and to healthy physical and cognitive development (Bento and Dias, 2017; Whitebread et al., 2017; Lai et al., 2018). Scholars such as Bundy (1997) and Cornelli Sanderson (2010) have noted that children’s approach to play, termed playfulness, is of particular significance for their creativity and daily play behaviors. Playfulness describes the quality of children’s play, children’s ability and willingness to engage in play, and their enjoyment in play (Barnett, 1991). Scholars have previously argued that playfulness refers to a relatively stable internal predisposition or personality trait that brings a playful quality to interactions (e.g., Lieberman, 1965, 1977; Trevlas et al., 2003). Recently, however, playfulness has been viewed as the expression of a capacity that can be promoted and encouraged by external factors in child–environment interactions (e.g., Bronson and Bundy, 2001; Cornelli Sanderson, 2010).

Studies have shown that high levels of playfulness in young children are associated with higher prosocial skills, higher levels of confidence and imagination, and more divergent thinking (Sicim Sevin, 2017; Barnett, 2018). Other recent studies have indicated that children’s playfulness predicts their future cognitive and social–emotional development and thus may contribute to better school readiness and formal school transitions (Fink et al., 2020; Fung and Chung, 2022; Waldman-Levi et al., 2022).

### 1.2 Assessment of children’s playfulness

Lieberman (1965, 1977) conducted initial research on playfulness by observing children at play. She observed individual differences in children’s spontaneity, sense of humor, and joyfulness, and she operationalized playfulness with five dimensions: (1) physical spontaneity describes the level of movement and coordination during play; (2) cognitive spontaneity describes the imaginary aspects of the child’s play and the child’s willingness to slip into different roles while playing; (3) social spontaneity describes the child’s flexibility in dealing with other children and the child’s willingness to initiate a play activity and to cooperate with others; (4) manifest joy describes the child’s ability to express joy and enthusiasm during play; and (5) a sense of humor describes the child’s ability to fool around, gently tease others, and joke with other children. The range of these five dimensions demonstrates the holistic nature of children’s play behavior.

To date, children’s playfulness has almost exclusively been assessed by proxy report from parents and/or professionals. Generally, this has been done either in questionnaire-based approaches using the CPS (Barnett, 1991; e.g., Trevlas et al., 2003; Yoon et al., 2014; Keleş and Yurt, 2017) or with external observations using the Test of Playfulness (ToP; Skard and Bundy, 2008, 2011; e.g., Pearton et al., 2014; Pinchover et al., 2016; Waldman-Levi et al., 2022). Barnett (1991) continued Lieberman’s research and developed the CPS: a reliable, valid, and easily used instrument to assess children’s playfulness from early to middle childhood from an adult perspective. The CPS consists of 23 items distributed over five subscales, described above as the five dimensions of playfulness and answered on a 5-point Likert scale (1 = does not sound at all like the child; 5 = sounds exactly like the child). In addition to the CPS, which assesses children’s playfulness as a general expression independent of situation, many studies used the ToP, which assesses children’s playfulness rather as behavior dependent on a concrete play situation. The ToP also assesses children’s playfulness in observations of 15-min play sequences with a multidimensional set of 33 items (Skard and Bundy, 2008, 2011).

Fink et al. (2020) developed the Child Self-Reported Playfulness Scale (CSRP), the first instrument to directly interview children aged 5 to 7 years about their playfulness. The CSRP is based on several established scales that assess children’s or adult’s playfulness (e.g., the CPS, Barnett, 1991; Adult Playfulness Trait Scale, Shen, 2010). The CSRP evaluates children’s global playfulness on a 10-item scale using hand puppets in a dichotomous response format. However, some authors have argued that strongly play-based procedures such as with puppets may lead children to focus more on the imagery than the verbal content (Marsh et al., 1998; Sturgess et al., 2002), and that pictures such as those used by Harter and Pike (1984) can be misleading as they sometimes have a different meaning for a child than for adults (Dockrell et al., 2000). Furthermore, no instrument currently assesses various dimensions of playfulness in self-report from children under the age of 5 years with a more differentiated response format than simple dichotomies.

### 1.3 Self-report assessments with young children

Active participation of young children is a fundamental principle in childhood research (Honig et al., 1999; Bamler et al., 2010; Heinzel, 2012). Studies have shown that even children at the age of 3 years are able to provide valid information about their experiences and skills (e.g., Marsh et al., 2002; Sturgess et al., 2002; Durbin, 2010; Müller et al., 2015). The first studies to directly interview children assessed the child’s self-concept, because only individuals themselves can provide insight into their own self-concept from their own perspectives. Several studies have examined children’s self-concept in social and academic domains (e.g., Harter and Pike, 1984; Marsh et al., 2002). The social self-concept describes how someone sees themselves through their acceptance by others and their abilities to engage in social interactions (Berndt and Burgy, 1996). This concept is particular important for understanding children’s playfulness, because playfulness involves children’s behavior and interactions with other children. Consequently, when children assess their own playfulness, they also consider their own experiences and perceptions when they engage in play and interact with others. Studies have indicated that children with higher levels of playfulness participate in more peer play (Rentzou, 2014; Fink et al., 2020), and this leads to better social development (Newton and Jenvey, 2011; Fung and Chung, 2022).

Children’s perspectives are unique and can only be reported by the children themselves (Sturgess et al., 2002; Danielson and Phelps, 2003). It cannot be assumed that adults’ statements accurately represent a child’s perspective (Mey, 2003). Moreover, children have an advantage in assessing their own behavior in various situations, including home, educational settings, and leisure activities, which adults often cannot do due to their specific roles in children’s lives. For instance, teachers’ perspectives are limited to institutional settings. The important influence of setting was demonstrated in a study by Christian (2012), which showed only a weak correlation between parents’ and teachers’ assessments of children’s playfulness.

Various studies in other fields, for example quality of life and physical activity habits, examined convergencies and discrepancies between children’s self-reports and parents’ and caregivers’ proxy reports. These studies generally showed significant but weak correlations between self- and proxy reports. This was found in studies with preschool children (e.g., Sotos-Prieto et al., 2015), but also school-aged children (e.g., Cremeens et al., 2006; Limbers et al., 2011; Shiroiwa et al., 2019). A study of the association between children’s self-reports and adult’s proxy reports on children’s social self-concept found no significant correlations (Engel, 2020). This was the case for both preschool and school-aged children.

### 1.4 Summary and research questions

In conclusion, children’s playfulness has mainly been assessed from the perspective of adults using questionnaires or external observation of children’s play behavior. To date, to the best of our knowledge, only one study has included children in the assessment of their self-reported playfulness, using the CSRP (Fink et al., 2020). However, this scale does not include 3- and 4-year-old children, provides only two response options, and assesses playfulness as a global construct. A dichotomous response format cannot adequately capture the nuances of children’s playfulness and is therefore less similar to the multidimensional CPS with five response options or to the ToP with four response options. We use a multidimensionally constructed scale to assess children’s playfulness in self-report. This approach enables us to map the construct of playfulness holistically from the children’s perspectives and thus develop a more detailed understanding of differences in children’s playfulness.

The aim of the present study was to adapt and validate the CPS (Barnett, 1991) as a self-report instrument for assessing the self-perceived playfulness of children between 3 and 8 years of age. The adapted and modified scale was designed to overcome the dichotomous nature of the response format of earlier studies (Fink et al., 2020), to account for the multidimensional nature of playfulness postulated by Lieberman (1965, 1977), and to address some of the potential challenges related to children’s tendency to focus more on imagery than verbal content in play-based approaches such as with hand puppets (Marsh et al., 1998; Sturgess et al., 2002).

This study addressed four research questions:

1. Can five distinguishable domains as postulated by Lieberman (1977) be identified in children’s self-report of playfulness?
2. Can the adapted and modified CPS scale in self-report for children demonstrate measurement invariance across different subgroups for age, gender, and language skills?
3. What is the relation between children’s self-reported playfulness and their self-reported social self-concept?
4. What is the relation between children’s self-reported playfulness and teacher’s proxy-reported children’s playfulness?

We hypothesized a strong association between children’s self-reported playfulness and their self-reported social self-concept, because children who are socially competent and accepted also tend to show higher levels of playful interactions and behaviors (H1) (Newton and Jenvey, 2011; Fink et al., 2020). Furthermore, we hypothesized from Christian’s (2012) study that there is no significant correlation between children’s self-reported playfulness and teacher’s proxy-reported children’s playfulness (H2). Due to the explorative character of the first two research questions, no hypotheses were formulated for them.

## 2 Method

### 2.1 Procedure

The present study was part of a project named “Playfulness in early childhood: A longitudinal study of individual and contextual determinants (Playful).” In this project, 81 teachers from 34 childcare centers and 47 kindergartens in 12 cantons in the German-speaking part of Switzerland gave their written consent to participate in the project. Childcare centers are privately funded and are generally open to children from infancy until they enter kindergarten. Kindergartens are part of the public school system in Switzerland for children between the ages of 4 and 7 years. When providing their consent, the teachers agreed to pass on information about the study to parents and, when parental consent was obtained, completed a questionnaire about each participating child in their group or class.

In total, 848 parents agreed to complete a questionnaire and granted permission to the research team to conduct an interview with their child at the childcare center or kindergarten. To take part in the child assessment, the child had to be at least 3 years old, to be present at the childcare center or kindergarten on the day of assessment, and to provide their verbal agreement to take part in the study. A total of 564 children met these criteria and participated in the present study. For these children, 540 (95.74%) teachers and 494 (87.59%) parents filled in a standardized online questionnaire that was administered with Survalyzer software.

The child assessment was conducted by trained research assistants. All research assistants underwent a half-day training session and were required to complete a pilot test with one child who did not participate in the study. The pilot test was videotaped, and feedback was provided to the research assistants to ensure standardized implementation of the child assessment.

All children were assessed individually, either in a separate room or a quiet area of their childcare center or kindergarten. The assessment lasted about 15 to 20 min per child and included questions on self-rated playfulness, self-rated social self-concept, and a language test. The assessment was conducted in Swiss German.

Teachers, parents, and children were informed that their participation was voluntary, that the data would be anonymized after survey, that these data would be used for scientific purposes only, and that withdrawal from the study was possible at any time without providing any reason.

### 2.2 Participants

Of the 564 children assessed, 266 were female (47.2%). The children were between 36 months and 106 months old (*M* = 66 months, *SD* = 13.71 months). Some 155 children attended a childcare center for an average of 2.5 days (*Min* = 0.5 days; *Max* = 5 days), and 409 children attended kindergarten on average for five mornings (*Min* = 2 mornings; *Max* = 5 mornings) and one afternoon (*Min* = no afternoon; *Max* = 4 afternoons).

Of the children, 70.5% were Swiss, 11% held dual citizenship of Swiss and another nationality, and 18.5% were of foreign nationality, mainly other European. In 57.5% of the families, the main language that was spoken was German, 29.8% spoke German and another language, and 12.9% did not speak German at home. Slightly more than half of the mothers (53.9%) and fathers (54.4%) had a degree from a university, university of applied sciences, or university of teacher education.

The majority of teachers were female (95%). The teachers were on average 37.5 years old (*SD* = 11.8 years) and had worked for an average of 13.6 years (*SD* = 10.5 years) as a teacher in a childcare center or kindergarten.

### 2.3 Instruments

#### 2.3.1 Teacher’s questionnaire

The teachers from the childcare centers and kindergartens assessed children’s playfulness for each child with the German version (Wustmann Seiler et al., 2021) of the Children’s Playfulness Scale (CPS; Barnett, 1991). The CPS contains 23 items assessed by the adults using a 5-point Likert scale (1 = does not sound at all like the child; 5 = sounds exactly like the child). The 23 items are distributed over five subscales: (1) physical spontaneity, containing four items (e.g., “The child’s movements are generally well-coordinated during play activities”); (2) cognitive spontaneity, containing four items (e.g., “The child invents his/her own games to play”); (3) social spontaneity, containing five items (e.g., “The child plays cooperatively with other children”); (4) manifest joy, containing five items (e.g., “The child expresses enjoyment during play”); and (5) sense of humor, containing five items (e.g., “The child enjoys joking with other children”). To construct the model in the same way as that for children (described below), a confirmatory factor analysis was conducted from the mean values of the five subscales. These five mean values were modelled as manifest indicators of the latent factor playfulness. Two error correlations were allowed, between social spontaneity and cognitive spontaneity and between cognitive spontaneity and manifest joy, to improve model fit. The model fitted the data sufficiently (χ^2^ [3] = 22.93, *p* < 0.001, CFI = 0.985; RMSEA = 0.081; SRMR = 0.021; *N* = 536), and the latent factor showed satisfactory internal consistency (McDonald’s Omega ω = 0.74).

#### 2.3.2 Child assessment

##### 2.3.2.1 Children’s playfulness

To assess children’s playfulness in self-report, we adapted the German version of the CPS (Wustmann Seiler et al., 2021) to use directly with children. We selected the three items in each subscale that showed the best psychometric properties in the study by Wustmann Seiler et al. (2021). We shortened the items to fit the attention and concentration span of children. We also slightly adapted the wording of the items for young children to make them easier for them to understand. For instance, the teachers’ item “the child initiates play with others” was adapted to the children’s item “do you like to invite other children to play with you?.” A comparison of all items can be found in Table 1. The response format was adapted according to Marsh et al. (1998) in a two-level response format. The children first responded to whether they liked or disliked doing something with either “yes, I like doing it” or “no, I do not like doing it.” They were presented with circles of paper in different colors to support the question visually. The children were asked to choose a green circle if the answer was yes and a blue circle if the answer was no. Although more intuitive, a red–green answer format was deliberately not chosen to avoid confusing any children with red–green color blindness. Secondly, children chose between a large and a small circle of the color they had chosen to indicate whether they liked doing it “very much,” with a large green circle or “just a little bit,” with a small green circle, or conversely that they “do not like doing it at all,” with a large blue circle, or “just a little bit,” with a small blue circle. As suggested by Harter and Pike (1984), this two-level response procedure was used to counteract an idealized self-representation of the children. It also ensured that the children used the entire response scale. To reduce complexity, no negative formulations were used (Marsh and Holmes, 1990). At the beginning of the assessment, we asked two sample questions that were unrelated to playfulness to familiarize the children with the response format (e.g., “Do you like eating spaghetti?”), and then proceeded to go through the 15 items of the scale (the 15 items were the same for all children, regardless of age). If a child did not understand a question, the question was supplemented with examples. To ensure standardization, predefined examples were used. For instance, if the child had difficulty to understand the question: “Do you like to stay longer with one activity during play?,” the clarifying statement was provided: “Do you like to stay longer with one activity during play instead of constantly changing the play activity?.” The feasibility of the response format and the comprehensibility of the wording was confirmed in a pilot test conducted in spring 2018 with 17 children attending childcare and kindergarten.

**Table 1:** The 15 CPS items in children’s self-report vs. the original 23 items (Barnett, 1991).

The 15 CPS items in children’s self-report	The original 23 items
**Social spontaneity**	
1. Do you like to take a leadership role when you are playing with other children?	1. The child assumes a leadership role when playing with others.
2. Do you like to ask other children to play with you?	2. The child initiates play with others.
3. Do you like to share playthings in the childcare center/kindergarten?	3. The child is willing to share playthings.
	4. The child responds easily to others’ approaches during play.
	5. The child plays cooperatively with other children.
**Cognitive spontaneity**	
4. Do you like to think of new things while playing such as a new game, new roles, or new rules?	6. The child invents his/her own games to play.
5. Do you like to stay longer with one activity during play?	7. The child stays with one activity rather than changes activity during play.
6. Do you like to play different character roles and dress up while playing?	8. The child assumes different character roles in play.
	9. The child uses unconventional objects in play.
**Physical spontaneity**	
7. Do you like to run and skip during play?	10. The child runs (skips, hops, jumps) a lot in play.
8. Do you like to play quietly without moving around too much such as sitting at a table and playing something?	11. The child prefers to be active rather than quiet in play.
9. When playing, do you like to move actively such as running, climbing, and jumping?	12. The child is physically active during play.
	13. The child’s movements are generally well-coordinated during play activities.
**Sense of humor**	
10. When playing with other children, do you like to fool around, such as with jokes and grimaces?	14. The child enjoys joking with other children.
11. Do you like to say funny things while you are playing?	15. The child tells funny stories.
12. When playing, do you like to tease the other children a bit by tickling them or hiding things from them?	16. The child gently teases others while at play.
	17. The child laughs at humorous stories.
	18. The child likes to clown around in play.
**Manifest joy**	
13. Do you like to talk while playing?	19. The child sings and talks while playing.
14. Do you like to play so intensely or concentratedly or focused that you do not get distracted*?*	20. The child shows enthusiasm during play.
15. Do you like to show your enjoyment when playing, for example, by smiling or laughing out loud?	21. The child expresses enjoyment during play.
	22. The child demonstrates exuberance during play.
	23. The child is restrained in expressing emotion during play.

Children’s Playfulness Scale in self-report was assessed on a 4-point Likert scale (1 = No, I do not like doing it; 4 = Yes, I like doing it), in proxy-reports on a 5-point Likert scale (1 = Does not sound at all like the child; 5 = Sounds exactly like the child).

##### 2.3.2.2 Social self-concept

Children were asked about their social self-concept using the social self-concept subscale, with seven items, from the Self-Concept Questionnaire for Children (*Selbstkonzeptfragebogen für Kinder*; Engel, 2020). The items were designed for children from 3 years with four response options (1 = very little, 2 = a little, 3 = quite a lot, 4 = a lot). Similarly, to the response procedure described for assessing children’s playfulness, blocks of different sizes were used to represent the response options visually in order to reduce the complexity for the children. Each question was answered in two steps; for example, (1) “Do other children like to play with you?” was followed by (2) “How much do the other children like to play with you?” After conducting a confirmatory factor analysis, two items were excluded due to low factor loadings (<0.3). The remaining 5 items loaded on the latent factor social self-concept with an acceptable model fit (χ^2^ [5] = 19.41, *p* < 0.01, CFI = 0.963; RMSEA = 0.066; SRMR = 0.033). Reliability was sufficient with a McDonalds Omega ω = 0.67 (*N* = 466).

##### 2.3.2.3 Language ability

To assess language ability, the language section of the Intelligence and Development Scales–Preschool (IDS-P; Grob et al., 2013) was used for children between the ages of 3 and 5:11 and the language section of the Intelligence and Development Scales (IDS; Grob et al., 2009) for children from 6 years. This combination allowed assessment of both active and passive language comprehension for all children. All raw values were converted into value equivalents with standard tables. The mean value equivalent of the passive and active language part was calculated as the language test score.

#### 2.3.3 Parent questionnaire

Parents provided information on demographic characteristics of the child and the family: child’s age and gender, family nationality and language, and parents’ level of education.

### 2.4 Analyses

Statistical analyses were conducted in R (R Core Team, 2022) using the lavaan package (Rosseel, 2012) to perform multiple-group factor analysis. To assess construct validity, a confirmatory factor analysis was calculated from the mean values of the five subscales of children’s playfulness in self-report. We modelled the five mean values as manifest indicators of the latent factor playfulness. Since each subscale consisted of only three items, no second-order model was computed, and consequently, no individual item analyses were conducted because there was no basis for excluding items. The generalization of the model was verified with invariance tests using multiple-group confirmatory factor analysis with gender, age, and language skills as analysis criteria. Gender was classified into two groups: male and female. Age was divided into under 5 years and over 5 years. Classification into these two groups was made because children from the age of 5 years begin to compare themselves with other children, and therefore their ideas about themselves become increasingly differentiated (Harter and Pike, 1984). Language skills were classified into three groups: below-average performance, average performance, and above-average performance. This classification was taken from the manual for IDS and IDS-P (Grob et al., 2009, 2013). To test invariance, four models were calculated. Model A examined configurational invariance. In this model, the loading pattern was the same in all groups, but parameters such as loadings, intercepts, and variance were allowed to differ between the groups. Model B tested metric invariance, and factor loadings were set equal for all groups. Model C tested scalar invariance. In this model, both factor loadings and item intercepts were set equal in all groups. Model D measured strict invariance, which means factor loadings, item intercepts, and residual variances were set equal for all groups (Chen, 2008; Hirschfeld and von Brachel, 2014). Because data were revealed not to be normally distributed (Shapiro–Wilk test, *p* < 0.001 for all items), we analyzed all models with a maximum likelihood estimator with robust standard errors. To indicate whether the fit of the more restrictive model did not differ significantly from the fit of the less restrictive model, a Chi-square difference test was used. Due to the robust maximum likelihood estimator, the difference test was checked with a Satorra-Bentler correction (Satorra and Bentler, 2001). Missing data were substituted using the full information maximum likelihood method. Because all children were grouped in childcare centers or kindergartens (*M* = 7 children; *Min =* 1 child; *Max* = 23 children), all calculations were performing with clustering (ICC_Teacher_ = 0.037; ICC_Children_ = 0.00).

## 3 Results

### 3.1 Descriptive results

Table 2 presents descriptive statistics for all five subscales and the total playfulness score in children’s self-report and in proxy report by teachers. In self-reports, the children scored the highest values on the physical spontaneity subscale (*M* = 3.44, *SD* = 0.60) and the lowest scores on the sense of humor subscale (*M* = 2.84, *SD* = 0.78). In the teachers’ proxy reports, the children scored the highest values on the manifest joy subscale (*M* = 4.13, *SD* = 0.66) and the lowest scores on the social spontaneity subscale (*M* = 3.53, *SD* = 0.76). All subscales of children’s playfulness scale in self-report correlated significantly positive to each other at a low to moderate level (*r* = 0.27–0.46). Intercorrelations of the subscales on teachers’ proxy reports were significantly positive at a moderate to high level (*r* = 0.43–0.67). All intercorrelations are reported in Table 2.

**Table 2:** Descriptive statistics and intercorrelations of all study variables.

		*N*	*M*	*SD*	ICC	1	2	3	4	5	6	7	8	9	10	11	12
1	Children’s playfulness (S)^1^	526	3.20	0.48	0.00	1											
2	Social spontaneity (S)	523	3.23	0.68	0.00	0.64***	1										
3	Cognitive spontaneity (S)	525	3.22	0.68	0.00	0.67***	0.46***	1									
4	Physical spontaneity (S)	523	3.44	0.60	0.00	0.62***	0.38***	0.41***	1								
5	Sense of humor (S)	523	2.84	0.78	0.01	0.48***	0.32***	0.32***	0.27***	1							
6	Manifest joy (S)	521	3.27	0.68	0.00	0.65***	0.40***	0.41***	0.45***	0.32***	1						
7	Social self-concept ^1^	466	3.39	0.70	0.02	0.54***	0.41***	0.28***	0.31***	0.07	0.41***	1					
8	Children’s playfulness (P)^1^	541	3.79	0.60	0.04	0.03	0.01	0.07	−0.06	0.02	0.07	0.16*	1				
9	Social spontaneity (P)	541	3.53	0.76	0.06	−0.01	−0.03	0.04	−0.06	−0.11*	0.09*	0.23***	0.65***	1			
10	Cognitive spontaneity (P)	541	3.82	0.76	0.01	0.05	−0.02	0.09*	0.02	−0.05	0.06	0.10^†^	0.63***	0.66***	1		
11	Physical spontaneity (P)	541	3.91	0.76	0.03	0.01	0.02	0.01	−0.05	0.02	0.03	0.11^†^	0.77***	0.43***	0.45***	1	
12	Sense of humor (P)	541	3.57	0.80	0.01	−0.05	−0.0.04	0.00	−0.09*	−0.01	0.00	0.11^†^	0.76***	0.54***	0.51***	0.61***	1
13	Manifest joy (P)	541	4.13	0.66	0.01	0.09^†^	0.04	0.10*	−0.01	0.08^†^	0.07	. 11^†^	0.84***	0.57***	0.65***	0.67***	0.61***

^1^latent model; (S) = children’s self-report; (P) = teachers’ proxy report; ^†^*p* < 0.10; ^*^p < 0.05; ^**^p < 0.01; ^***^p < 0.001.

### 3.2 Confirmatory factor analysis

To test the factor structure using confirmatory factor analysis, the five subscales were modelled on the latent factor playfulness. The model fitted the data well (χ^2^ [5] = 5.24; *p* = 0.388; CFI = 0.999; RMSEA = 0.010; SRMR = 0.016). All subscales showed sufficient factor loadings on the latent factor playfulness (>0.40), and internal consistency was satisfied with a McDonalds Omega ω = 0.74.

### 3.3 Multiple-group invariance testing

Multiple-group invariance testing was performed for four models—configural, metric, scalar, and strict—grouped by gender, age, and language skills. Table 3 illustrates fit indices for all models. Each of the individual models showed acceptable model fit.

**Table 3:** Multiple-group invariance testing grouped by gender, age, and language skills.

Model across gender	Compared Model	χ*^2^(df)*	ΔSBχ*^2^ (*Δ*df)*	CFI	RMSEA	SRMR
A: Configural invariance		8.264 (10)		1.000	0.000	0.017
B: Full metric invariance	A	16.899 (14)	+9.321 (4)	0.993	0.028	0.044
C: Full scalar invariance	B	25.149 (18)	+ 8.527 (4)	0.982	0.039	0.050
D: Full strict invariance	C	28.123 (23)	+ 3.300 (5)	0.987	0.029	0.056
Model across age						
A: Configural invariance		8.832 (10)		1.000	0.000	0.020
B: Full metric invariance	A	11.731 (14)	+2.830 (4)	1.000	0.000	0.030
C: Full scalar invariance	B	17.924 (18)	+6.755 (4)	1.000	0.000	0.035
D: Full strict invariance	C	26.079 (23)	+8.119 (5)	0.992	0.023	0.046
Model across language skills						
A: Configural invariance		10.012 (15)		1.000	0.000	0.022
B: Full metric invariance	A	13.024 (23)	+2.642 (8)	1.000	0.000	0.031
C: Full scalar invariance	B	32.791 (31)	+21.175 (8)**	0.994	0.019	0.049
D: Partial scalar invariance (except PS)	B	23.685 (29)	+11.644 (6)	1.000	0.000	0.043
E: Partial strict invariance (except PS)	D	31.039 (39)	+7.399 (10)	1.000	0.000	0.054

PS, physical spontaneity; χ^2^, Satorra-Bentler scaled Chi-square; df, degrees of freedom; Δ, difference value; CFI, comparative fit index; RMSEA, root mean square error of approximation; SRMR, standardized root mean square residual; **p < 0.01.

We found no significant differences between the four models grouped by gender and age. This means that the fit of the more restrictive models did not differ significantly from the fit of the less restrictive models. Thus, we were able to demonstrate full strict invariance across gender and age.

For invariance testing across language skills, we found a significant difference between the metric and scalar models (ΔSBχ2 (8) = 21.18, *p* < 0.01). Therefore, partial scalar invariance was tested (Hirschfeld and von Brachel, 2014) by considering modification indices. The highest values of modification were found in the physical spontaneity subscale. Hence, the physical spontaneity subscale was freezed from all further steps. By freezing this subscale, we were able to show partial strict invariance for language skills.

### 3.4 Association between children’s self-reported playfulness and social self-concept

To test for convergent validity, we examined correlations between children’s self-reported playfulness and self-reported social self-concept. As reported in Table 2, we found a significant high correlation between children’s playfulness and children’s social self-concept (*r* = 0.54, *p* < 0.001). For the social spontaneity, cognitive spontaneity, physical spontaneity, and manifest joy subscales, we found a moderate significant correlation with children’s social self-concept (*r* = 0.28–0.41). Only between the sense of humor subscale and social self-concept was no significant correlation identified.

### 3.5 Association between children’s self-reported playfulness and teachers’ proxy-reported playfulness in children

The association between self- and proxy assessment was assessed by calculating correlations between children’s self-reports and teachers’ proxy reports. As presented in Table 2, no significant correlation was found between the total playfulness score in children’s self-reports and the total playfulness score in teacher’s proxy reports (*r* = 0.03, *p =* 0.92). We could only find a significant positive correlation for cognitive spontaneity (*r* = 0.09, *p <* 0.05) between the corresponding subscales.

## 4 Discussion

The present study aimed to develop and validate the Children’s Playfulness Scale (CPS; Barnett, 1991) as a self-report instrument for children aged 3 years and older with a multidimensional structure and a comparable response format to that used in proxy reports by parents and teachers. The study included 564 children from 81 childcare centers and kindergartens in Switzerland. To test for factorial validity of the self-report scale, multiple-group invariance tests were computed grouped by gender, age, and language skills. In addition, convergent validity was tested by examining relations to children’s self-reported social self-concept, and relations to teachers’ proxy reports of children’s playfulness were explored. Results showed that the multidimensional structure of children’s playfulness was reliably assessable even from the self-report of children of 3 years of age, and evidence confirmed acceptable measurement invariance and convergent validity. The present study is therefore the first to present a differentiated instrument for assessing children’s playfulness from the perspectives of the young children themselves.

### 4.1 The children’s playfulness scale in self-report

Our first research question was concerned with whether the five distinguishable domains as postulated by Lieberman (1977) be identified in children’s self-report of playfulness. Here, we were able to show that the five manifest subscales social spontaneity, cognitive spontaneity, physical spontaneity, sense of humor, and manifest joy, loaded significantly on the latent factor playfulness. This indicates that children’s self-assessment associated all five dimensions with the construct of playfulness. The second research question was addressed by measuring invariance, and factor structure was shown to be stable across gender, age, and language skills. Analysis of multiple-group comparison indicated full strict invariance for age and gender. For language performance, partial strict invariance was found. This indicated that the self-report instrument was not dependent on gender, age, or language skills. Since no differences were found between boys and girls, age, or language skills, the self-reported CPS can be generalized for children with similar sample characteristics.

As Hypothesis 1 predicted, convergent validity was confirmed by high correlations between children’s self-reported playfulness and children’s self-reported social self-concept. The highest correlation was found between social self-concept and two subscales, social spontaneity and sense of humor. This corresponds to the two subscales described by Lieberman (1965, 1977) as having the most social associations with playfulness.

Overall, we were able to demonstrate that children as young as 3 years old are able to provide information about their own playfulness, this is evidence by the fact, that we were able to proof scalar invariance across groups. These results are consistent with other studies that have shown that children can reliably report on themselves at a very young age (e.g., Durbin, 2010; Müller et al., 2015; Engel, 2020). In addition, the study was able to provide initial indications of convergent validity with the connection between the children’s playfulness scale in self-report and the construct of social self-concept.

### 4.2 Discrepancies between children’s self-report and teachers’ proxy-report

As postulated in Hypothesis 2, we found no relation between children’s playfulness in self-reports and children’s playfulness in proxy reports by teachers. This indicated that children rate their own playfulness differently than teachers rate the same children’s playfulness. These findings are consistent with other studies that have investigated agreement and discrepancies between children’s self-report and adults’ proxy report on various topics. For example, Engel (2020) found no significant correlation between children’s self-report social self-concept and adults’ proxy report of children’s social self-concept.

These results may be explained by various aspects of potential perceptual or methodological bias. First, children observe themselves in more environmental settings than adults (Danielson and Phelps, 2003; Sotos-Prieto et al., 2015). In our study, teachers observed the children only in the childcare center or kindergarten, whereas children also observed themselves at home, outside in the playground, and during various leisure activities such as sports. Second, the difference may be explained by children’s development. Young children tend to overestimate themselves, which can lead to an unrealistically positive assessment of themselves (Harter, 2012). In contrast, the responses of older children are subject to social desirability. For instance, older children may report that they refrain to slightly tease other children, even if they desire to, due to the assumption adults would disapprove. Conversely, younger children may say, that they like to tell funny things while playing, even though they lack the ability to do so effectively. Third, the reason might be methodological, arising because children’s assessment of playfulness was conducted differently from those of teachers. The children’s assessment was face-to-face, with four response options for each item, whereas teachers’ assessment was conducted with an online questionnaire and five response options. In addition, the adult version contained 23 items and the child version only 15 items, and these are not identically worded. The reduction was made to fit the child version within young children’s limited attention and concentration spans. Fourth, children live in the here and now and therefore provide information about their playfulness as they perceive it at the very moment in which they are asked. As a result, their responses on that specific day might be affected by various factors, such as a quarrel or losing a game. In contrast, teachers were likely to provide a more general evaluation of the child’s playfulness. Fifth, children’s play is the child’s main activity and field of learning. However, it can sometimes be difficult for adults to recognize what a child wants to achieve with their play. Thus, adults have a different view of children’s play than children, which in turn can affect the assessment of children’s playfulness. Finally, teachers are professionally trained and compare each specific child with various other children. Children are less likely to compare themselves with other children or only with a limited number of children they know well.

However, there is also the possibility that children do indeed differ from adults in the way they understand playfulness (Mey, 2003). For example, children sometimes identify other aspects of their play as important compared to adults or common scientific definition criteria of play (Barnett, 2013).

### 4.3 Future research and limitations

This study is the first to develop and validate an instrument for the self-assessment of children’s playfulness in children aged 3 years and older. Nevertheless, the study has some limitations.

First, the sample is representative of the educational level of parents in the general population, but not for the nationality of the children involved or for attendance at a childcare center: In our study, 53.90% of mothers and 54.42% of fathers had a university degree. This corresponds to the current education level of 25- to 45-year-olds in Switzerland (Bundesamt für Statistik, 2023a). However, the percentage of Swiss children in our study (81.50%) is higher than in the total population of children in Switzerland (73.93%) between 3 and 8 years of age (Bundesamt für Statistik, 2023b). It should also be considered that the study design included only children that attended a childcare center or kindergarten. However, in Switzerland, about 65.5% of 3-year-olds and some 4-year-olds do not attend childcare center or kindergarten (Bundesamt für Statistik, 2021). Children in childcare center or kindergartens may have more experience of playing in social groups, solving conflict situations with peers, and understanding social norms and rules. These interactions that children have in childcare centers and kindergartens are an important part of their social and psychological development at this age. Hence, attending childcare centers and kindergartens is a crucial investment in children’s social and psychological development, preparing them for a successful future in various social contexts. Therefore, the results may not be transferable to children who do not attend childcare center or kindergarten because they have not had such experiences. The results are thus primarily applicable to Swiss children attending childcare or kindergarten. Second, no independent assessment of children’s playfulness was included as an additional validation criterion, for example, through video or live observations using an observation scale such as the Test of Playfulness (Skard and Bundy, 2008, 2011). These additional data may be useful to better understand discrepancies between children’s self-reports and adults’ proxy reports of children’s playfulness and could be included in further studies. Third, children were only interviewed at one time point: no test–retest validation was included. In addition, measurement invariance over time is required to demonstrate a developmental perspective. Longitudinal data would enable researchers to determine whether children’s playfulness changes over time or whether it is a stable disposition or trait from the children’s perspectives. Fourth, because each manifest subscale consisted of only three items, no items could be excluded, or individual subscales would consist of only one or two items. Therefore, no individual item analyses could be performed. In addition, the second-order quasifactor playfulness was of particular interest, so we demonstrated that the five manifest subscales loaded on the latent factor playfulness. However, we were unable to prove that the five subscales were first-order distinguishable factors. Further studies should test the entire 23 items from the CPS in children’s self-report to model a real second-order model. Fifth, we provided convergent validity by examining the connection between the children’s playfulness scale in self-report and their social self-concept, which reflects their personal perceptions of social interactions. Further studies could explore the association between children’s self-reported playfulness and their social competences in a more specific sense to more fully demonstrate convergent validity. Because playfulness encompasses cognitive, emotional, and physical dimensions in addition to social skills, further studies may clarify what relationships exist between these developmental domains and the child’s self-reported playfulness.

### 4.4 Implications

The study findings have implications for both research and practice. For research purposes, the present study demonstrated that children from the age of 3 years can be reliably and directly asked about their own playfulness. Thus, in addition to the adult perspective, children’s perspectives should be included in studies, and children can thus actively participate in shaping knowledge about themselves. In practice, the findings emphasize that children do have their own perspectives on the world. It is worthwhile for adults to consider children’s perspectives in their actions. It also opens opportunities for adults to reflect on playfulness together with the child, thus promoting their metacognition on a topic that is highly relevant to them and very close to their everyday experience.

### 4.5 Conclusion

Overall, the adapted version of the Children’s Playfulness Scale by Barnett (1991) is an adequate instrument for assessing children’s playfulness in self-report for children of 3 years and older. In agreement with other studies, we found no significant relation between children’s self-report and teacher’s proxy report. This discrepancy highlights the importance of including children’s own perspectives in research. Young children should be viewed as experts on themselves because they can provide us with new and relevant information. However, successfully collecting data directly from young children requires specific expertise both in interviewing young children and in the field of early childhood education and child psychology.

## Data availability statement

The raw data supporting the conclusions of this article will be made available by the authors, without undue reservation.

## Ethics statement

The studies involving humans were approved by Ethics Committee of the Faculty of Philosophy of the University of Zurich. The studies were conducted in accordance with the local legislation and institutional requirements. Written informed consent for participation in this study was provided by the participants’ legal guardians/next of kin.

## Author contributions

ID: Data curation, Formal analysis, Investigation, Writing – original draft. CR: Data curation, Investigation, Writing – review & editing. CW: Conceptualization, Data curation, Formal analysis, Funding acquisition, Investigation, Writing – review & editing, Methodology, Project administration, Supervision. PL: Conceptualization, Funding acquisition, Writing – review & editing, Methodology, Supervision.

## References

[ref1] Bamler V. Werner J. Wustmann C. (2010). *Lehrbuch* Kindheitsforschung: Grundlagen, Zugänge und Methoden [textbook childhood studies: Foundations, approaches and methods]. Turin: Juventa.

[ref2] Barnett L. (1991). The playful child: Measurment of a dispisition to play. Play Cult. 4, 51–74.

[ref3] Barnett L. (2013). Children’s perceptions of their play: scale development and validation. Child Dev. Res. 2013, 1–18. doi: 10.1155/2013/284741

[ref4] Barnett L. (2018). The education of playful boys: class clowns in the classroom. Front. Psychol. 9:232. doi: 10.3389/fpsyg.2018.00232, PMID: 29545760 PMC5839094

[ref5] Bento G. Dias G. (2017). The importance of outdoor play for young children’s healthy development. Porto Biomed. J. 2, 157–160. doi: 10.1016/j.pbj.2017.03.003, PMID: 32258612 PMC6806863

[ref1001] Berndt T. J. Burgy L. (1996). “Social self-concept,” in Handbook of self-concept: Developmental, social, and clinical considerations. John Wiley & Sons. 171–2090.

[ref6] Bronson M. R. Bundy A. C. (2001). A correlational study of a test of playfulness and a test of environmental supportiveness for play. Occup. Ther. J. Res. 21, 241–259. doi: 10.1177/153944920102100403

[ref7] Bundesamt für Statistik. (2021). Familien in der Schweiz. Statistischer Bericht 2021 [Families in Switzerland. Statistical Report 2021]. Available at: https://www.bfs.admin.ch/bfs/de/home/statistiken/bevoelkerung/familien/familienergaenzende-kinderbetreuung.assetdetail.17084546.html

[ref8] Bundesamt für Statistik. (2023a). Bildungsstand der ständigen Wohnbevölkerung nach Arbeitsmarktstatus, Geschlecht, Nationalität, Altersgruppen und Familientyp [educational level of the permanent resident population by labour market status, gender, nationality, age groups and family type]. https://www.bfs.admin.ch/bfs/de/home/statistiken/arbeit-erwerb/erwerbstaetigkeit-arbeitszeit/merkmale-arbeitskraefte/bildungsniveau.assetdetail.26505533.html

[ref9] Bundesamt für Statistik. (2023b). Ständige Wohnbevölkerung nach Alter, Geschlecht und Staatsangehörigkeitskategorie, 2010–2022 [Permanent resident population by age, sex and citizenship category, 2010–2022]. https://www.bfs.admin.ch/bfs/de/home/statistiken/bevoelkerung/stand-entwicklung/alter.assetdetail.26565150.html

[ref10] Bundy A. C. (1997). “Play and playfulness: what to look for” in Play in occupational therapy for children. eds. Parham D. Fazio L. (Maryland Heights, MI: Mosby), 52–66.

[ref11] Chen F. F. (2008). What happens if we compare chopsticks with forks? The impact of making inappropriate comparisons in cross-cultural research. J. Pers. Soc. Psychol. 95, 1005–1018. doi: 10.1037/a0013193, PMID: 18954190

[ref12] Christian K. M. (2012). The construct of playfulness: Relationships with adaptive behaviors, humor, and early play ability – ProQuest. Available at: https://www.proquest.com/openview/eb6f7ca2a3b5d6e25c8c57c89285c161/1?pq-origsite=gscholar&cbl=18750

[ref13] Cornelli Sanderson R. (2010). Towards a new measure of playfulness: The capacity to fully and freely engage in play [Loyola University Chicago]. Available at: http://ecommons.luc.edu/luc_diss/232

[ref14] Cremeens J. Eiser C. Blades M. (2006). Factors influencing agreement between child self-report and parent proxy-reports on the pediatric quality of life inventory™ 4.0 (PedsQL™) generic core scales. Health Qual. Life Outcomes 4:58. doi: 10.1186/1477-7525-4-58, PMID: 16942613 PMC1564004

[ref15] Danielson C. K. Phelps C. R. (2003). The assessment of children’s social skills through self-report: a potential screening instrument for classroom use. Meas. Eval. Couns. Dev. 35, 218–229. doi: 10.1080/07481756.2003.12069068

[ref16] Dockrell J. Lewis A. Lindsay G. (2000). “Researching children’s perspectives: a psychological dimension” in Researching children’s perspectives. eds. Lewis A. Lindsay G. (Maidenhead: Open University Press), 46–58.

[ref17] Durbin C. E. (2010). Validity of young children’s self-reports of their emotion in response to structured laboratory tasks. Emotion 10, 519–535. doi: 10.1037/a0019008, PMID: 20677869

[ref18] Engel E.-M. (2020). Der Selbstkonzeptfragebogen für kinder (SKF). Entwicklung, Anwendung und psychometrische Überprüfung. Materialien zur Frühpädagogik band 17 [the self-concept questionnaire for children (SKF). Development, application and psychometric testing. Materials on early childhood education 17]. Zentrum für Kinder- und Jugendforschung

[ref19] Fink E. Mareva S. Gibson J. L. (2020). Dispositional playfulness in young children: a cross-sectional and longitudinal examination of the psychometric properties of a new child self-reported playfulness scale and associations with social behavior. Infant Child Dev. 29, 1–25. doi: 10.1002/icd.2181

[ref20] Fung W. K. Chung K. K. H. (2022). Parental play supportiveness and kindergartners’ peer problems: Children’s playfulness as a potential mediator. Soc. Dev. 31, 1126–1137. doi: 10.1111/sode.12603

[ref21] Ginsburg K. R. (2007). The importance of play in promoting healthy child development and maintaining strong parent-child bonds. Pediatrics 119, 182–191. doi: 10.1542/peds.2006-2697 17200287

[ref22] Grob A. Meyer C. S. Hagmann-von Arx P. (2009). Intelligence and Development Scales (IDS). Intelligenz- und Entwicklungsskalen für Kinder von 5–10 Jahren. Bern: Verlag Hans Huber.

[ref23] Grob A. Reimann G. Gut J. Frischknecht M.-C. (2013). Intelligence and Development Scales – Preschool (IDS-P). Intelligenz- und Entwicklungsskalen für das Vorschulalter. Bern: Verlag Hans Huber.

[ref1002] Harter S. (2012). Self-perception profiles for children: Manual and questionnaires (Grades 3-8). University of Denver.

[ref24] Harter S. Pike R. (1984). The pictorial scale of perceived competence and social acceptance for young children. Child Dev. 55, 1969–1982. doi: 10.2307/1129772 6525886

[ref25] Heinzel F. (2012). Methoden der Kindheitsforschung: Ein Überblick über Forschungszugänge zur kindlichen Perspektive [childhood research methods: An overview of research approaches to the child perspective]. 2nd Edn. Weinheim: Beltz.

[ref26] Hirschfeld G. von Brachel R. (2014). Improving multiple-group confirmatory factor analysis in R – A tutorial in measurement invariance with continuous and ordinal indicators. Pract. Assess. Res. Eval. 19:7. doi: 10.7275/QAZY-2946

[ref27] Honig M.-S. Lange A. Leu H. R. (1999). Aus der Perspektive von Kindern? Zur Methodologie der Kindheitsforschung [from the perspective of children? On the methodology of childhood research]. Weinheim: Juventa.

[ref28] Keleş S. Yurt Ö. (2017). An investigation of playfulness of pre-school children in Turkey. Early Child Dev. Care 187, 1372–1387. doi: 10.1080/03004430.2016.1169531

[ref29] Lai N. K. Ang T. F. Por L. Y. Liew C. S. (2018). The impact of play on child development—a literature review. Eur. Early Child. Educ. Res. J. 26, 625–643. doi: 10.1080/1350293X.2018.1522479

[ref30] Lieberman J. N. (1965). Playfulness and divergent thinking: an investigation of their relationship at the kindergarten level. J. Genet. Psychol. 107, 219–224. doi: 10.1080/00221325.1965.10533661, PMID: 5852592

[ref31] Lieberman J. N. (1977). Playfulness. Its relationship to imagination and creativity. Cambridge, MA: Academic Press.

[ref32] Lillard A. S. Lerner M. D. Hopkins E. J. Dore R. A. Smith E. D. Palmquist C. M. (2013). The impact of pretend play on children’s development: a review of the evidence. Psychol. Bull. 139, 1–34. doi: 10.1037/a0029321, PMID: 22905949

[ref33] Limbers C. A. Ripperger-Suhler J. Heffer R. W. Varni J. W. (2011). Patient-reported pediatric quality of life inventory™ 4.0 generic core scales in pediatric patients with attention-deficit/hyperactivity disorder and comorbid psychiatric disorders: feasibility, reliability, and validity. Value Health 14, 521–530. doi: 10.1016/j.jval.2010.10.031, PMID: 21315637

[ref34] Marsh H. W. Craven R. G. Debus R. (1998). Structure, stability, and development of young children’s self-concepts: a multicohort-multioccasion-study. Child Dev. 69, 1030–1053. PMID: 9768485

[ref35] Marsh H. W. Ellis L. A. Craven R. G. (2002). How do preschool children feel about themselves? Unraveling measurement and multidimensional self-concept structure. Dev. Psychol. 38, 376–393. doi: 10.1037/0012-1649.38.3.376, PMID: 12005381

[ref36] Marsh H. W. Holmes I. W. M. (1990). Multidimensional self-concepts: construct validation of responses by children. Am. Educ. Res. J. 27, 89–117. JSTOR. doi: 10.2307/1163070

[ref37] Mey G. (2003). Zugänge zur kindlichen Perspektive. Methoden der Kindheitsforschung [approaches to the child perspective. Childhood research methods]. Trier: Leibniz Institut für Psychologie (ZPID).

[ref38] Müller E. Wustmann Seiler C. Perren S. Simoni H. (2015). Young children’s self-perceived ability: development, factor structure and initial validation of a self-report instrument for preschoolers. J. Psychopathol. Behav. Assess. 37, 256–273. doi: 10.1007/s10862-014-9447-9

[ref39] Newton E. Jenvey V. (2011). Play and theory of mind: associations with social competence in young children. Early Child Dev. Care 181, 761–773. doi: 10.1080/03004430.2010.486898

[ref40] Nilsson M. Ferholt B. Lecusay R. (2018). ‘The playing-exploring child’: Reconceptualizing the relationship between play and learning in early childhood education. Contemp. Issues Early Child. 19, 231–245. doi: 10.1177/1463949117710800

[ref41] Pearton J. L. Ramugondo E. Cloete L. Cordier R. (2014). Playfulness and prenatal alcohol exposure: a comparative study. Aust. Occup. Ther. J. 61, 259–267. doi: 10.1111/1440-1630.12118, PMID: 24547920

[ref42] Piaget J. (1976). “Piaget’s theory” in Piaget and his school. eds. Inhelder B. Chipman H. H. Zwingmann C. (Berlin Heidelberg: Springer), 11–23.

[ref43] Pinchover S. (2017). The relation between teachers’ and children’s playfulness: a pilot study. Front. Psychol. 8:2214. doi: 10.3389/fpsyg.2017.02214, PMID: 29312069 PMC5742179

[ref44] Pinchover S. Shulman C. Bundy A. (2016). A comparison of playfulness of young children with and without autism spectrum disorder in interactions with their mothers and teachers. Early Child Dev. Care 186, 1893–1906. doi: 10.1080/03004430.2015.1136622

[ref45] R Core Team. (2022). R: A language and environment for statistical computing. R Foundation for Statistical Computing. Available at: https://www.r-project.org/

[ref46] Rentzou K. (2014). Preschool children’s social and nonsocial play behaviours. Measurement and correlations with children’s playfulness, behaviour problems and demographic characteristics. Early Child Dev. Care 184, 633–647. doi: 10.1080/03004430.2013.806496

[ref47] Rosseel Y. (2012). Lavaan: an R package for structural equation modeling. J. Stat. Softw. 48, 1–36. doi: 10.18637/jss.v048.i02

[ref48] Ruckser-Scherb R. (2010). Die Beziehung von Spielfähigkeit und Effektivität des coping bei Kindergartenkindern im Alter von 4—6 Jahren [the relationship of play ability and coping effectiveness in kindergarten children aged 4-6 years]. Ergoscience 5, 91–98. doi: 10.1055/s-0029-1245547

[ref49] Satorra A. Bentler P. M. (2001). A scaled difference chi-square test statistic for moment structure analysis. Psychometrika 66, 507–514. doi: 10.1007/BF02296192 PMC2905175 20640194

[ref50] Saunders I. Sayer M. Goodale A. (1999). The relationship between playfulness and coping in preschool children: a pilot study. Am. J. Occup. Ther. 53, 221–226. doi: 10.5014/ajot.53.2.221, PMID: 10200846

[ref51] Shen X. (2010). Adult playfulness as a personality trait: its conceptualization, measurement, and relationship to psychological well-being. The Pennsylvania State University.

[ref52] Shiroiwa T. Fukuda T. Shimozuma K. (2019). Psychometric properties of the Japanese version of the EQ-5D-Y by self-report and proxy-report: reliability and construct validity. Qual. Life Res. 28, 3093–3105. doi: 10.1007/s11136-019-02238-1 31243620 PMC6803591

[ref53] Sicim Sevin B. (2017). Investigating the association between playfulness, environment and social skills of preschool children. Ankara: Middle East Technical University.

[ref54] Skard G. Bundy A. C. (2008). “Test of playfulness” in Play in occupational therapy for children. eds. Parham L. D. Fazio L. S.. 2nd ed (Maryland Heights, MI: Mosby), 71–93.

[ref55] Skard G. Bundy A. C. (2011). Test of Playfulness (ToP)—Test zur Spielfähigkeit. Deutsche Übersetzung: Barbara und Jürgen Dehnhardt. Idstein: Schulz-Kirchner Verlag.

[ref56] Sotos-Prieto M. Santos-Beneit G. Pocock S. Redondo J. Fuster V. Peñalvo J. L. (2015). Parental and self-reported dietary and physical activity habits in pre-school children and their socio-economic determinants. Public Health Nutr. 18, 275–285. doi: 10.1017/S1368980014000330, PMID: 24698168 PMC10271003

[ref57] Sturgess J. Rodger S. Ozanne A. (2002). A review of the use of self-report assessment with young children. Br. J. Occup. Ther. 65, 108–116. doi: 10.1177/030802260206500302

[ref58] Trevlas E. Matsouka O. Zachopoulou E. (2003). Relationship between playfulness and motor creativity in preschool children. Early Child Dev. Care 173, 535–543. doi: 10.1080/0300443032000070482

[ref59] Vygotsky L. S. (1967). Play and its role in the mental development of the child. Sov. Psychol. 5, 6–18. doi: 10.2753/RPO1061-040505036

[ref60] Waldman-Levi A. Bundy A. Katz N. (2015). Playfulness and interaction: an exploratory study of past and current exposure to domestic violence. OTJR 35, 89–94. doi: 10.1177/1539449214561762, PMID: 26460471

[ref61] Waldman-Levi A. Bundy A. Shai D. (2022). Cognition mediates playfulness development in early childhood: a longitudinal study of typically developing children. Am. J. Occup. Ther. 76:7605205020. doi: 10.5014/ajot.2022.049120, PMID: 35947034

[ref62] Whitebread D. Neale D. Jensen H. Liu C. Solis S. L. Hopkins E.. (2017). The role of play in children’s development: A review of the evidence. Billund: The LEGO Foundation.

[ref63] Wustmann Seiler C. Lannen P. Duss I. Sticca F. (2021). Mitspielen, (An)Leiten, Unbeteiligt sein?: Zusammenhänge kindlicher und elterlicher Playfulness: Eine Pilotstudie [Playing along, leading, being uninvolved? Parental play guidance and children’s playfulness]. Frühe Bildung 10, 161–168. doi: 10.1026/2191-9186/a000526

[ref64] Yoon T.-H. Cha T.-H. Lee J.-S. (2014). The affecting factor on the children’s playfulness in South Korea. Int. J. Bio Sci. Bio Tech. 6, 23–32. doi: 10.14257/ijbsbt.2014.6.5.03

[ref65] Zosh J. M. Hopkins E. J. Jensen H. Liu C. Neale D. Hirsh-Pasek K.. (2017). Learning through play: A review of the evidence. Billund: The LEGO Foundation.

